# Isolated Intramedullary Thoracic Spinal Sarcoidosis: A Case Report and Review of the Literature

**DOI:** 10.7759/cureus.48375

**Published:** 2023-11-06

**Authors:** Caren M Stuebe, Jose M Soto, Yuan Shan, Frank Harris

**Affiliations:** 1 Neurosurgery, Texas A&M School of Medicine, Temple, USA; 2 Neurosurgery, Baylor Scott & White Medical Center - Temple, Temple, USA; 3 Pathology, Baylor Scott & White Medical Center - Temple, Temple, USA

**Keywords:** thoracic spine, intramedullary, sarcoidosis, literature review, case report

## Abstract

Sarcoidosis is a multisystemic inflammatory granulomatosis disease that rarely involves the central nervous system (CNS) and is even more so rarely isolated to the intramedullary thoracic spine. In isolated CNS sarcoidosis cases, surgical treatment is debated. We present here a case report and literature review on intramedullary thoracic spine sarcoidosis to evaluate potential portents of spine involvement and indications for surgical intervention. A 47-year-old female with a prior history of renal cell carcinoma presented with a week-long history of urinary retention and bilateral lower extremity numbness, and a 24-hour history of left lower extremity (LLE) weakness with saddle anesthesia. Magnetic resonance imaging demonstrated a syrinx spanning the spinal cord to the conus medullaris and a contrast-enhancing, expansile intramedullary thoracic lesion at T6-T7 with a non-enhancing, cystic right paraspinal lesion at T5. Given the patient’s history of a kidney neoplasm, a metastatic work-up was completed. Biopsy of the T5 lesion was consistent with endometriosis. The patient underwent a T6-8 laminectomy with excisional biopsy and gross total resection of the intramedullary mass. Initial pathology was notable for lymphohistiocytic infiltrate with coagulative necrosis and rare multinucleated giant cells. At the one-month follow-up, the patient had improving LLE weakness and continued impairment of gait, balance, and coordination, but her symptoms of urinary retention, paresthesia, and numbness were resolved. Final pathology supported a diagnosis of sarcoidosis. At the three-month follow-up, the patient reported intermittent surgical site pain, but no other symptoms. She is followed up by her primary care consultant for symptom management and recurrence monitoring. Apart from the presented case, only one case of isolated intramedullary thoracic spine sarcoidosis was identified in the literature. The only case, of both review and presented, without significant symptom improvement did not undergo surgery. The available literature is limited; however, early surgical intervention may be indicated in isolated thoracic spine sarcoidosis.

## Introduction

Sarcoidosis is a multisystemic inflammatory granulomatosis disease that typically presents with vague systemic symptoms, including pain, arthritis, skin lesions, weight loss, fatigue, and dyspnea [1,2]. On pathology, sarcoidosis is characterized by non-caseating granulomas on low-power microscopy and Langhans giant cells surrounded by epithelioid cells and lymphocytes on high-power microscopy [3]. Sarcoidosis of the central nervous system (CNS), termed neurosarcoidosis, is seen in up to 10% of patients, though involvement of the spinal cord specifically is still rare, seen in only 6%-8% of sarcoidosis patients [4-7].

Spinal sarcoidosis lesions can present as intramedullary (35%), extramedullary intradural (35%), a combination of both (23%), or extradurally (7%) [8]. In addition to its relative rarity, intramedullary spinal sarcoidosis is characterized by nonspecific clinical and imaging features that can be misleading, causing clinicians to mistake spinal sarcoidosis for more common neurological diagnoses. Thus, while magnetic resonance imaging (MRI) can assist in establishing a diagnosis, surgical biopsy with pathological examination is still the golden standard. Early interventions, such as first-line corticosteroids or second-line immunosuppressants, especially before signs of spinal cord atrophy, have been shown to improve outcomes [9-11].

Here, we present a case of and systematically review the literature on intermedullary thoracic spinal sarcoidosis, focusing on systemic versus isolated involvement, symptomatology, and treatment outcomes.

## Case presentation

A 47-year-old female presented to the emergency department in June 2022 with a week-long history of urinary retention and bilateral lower extremity (BLE) numbness and a 24-hour history of left lower extremity (LLE) weakness (2/5 strength) with saddle anesthesia. Fifteen years prior, the patient had a right nephrectomy, chemotherapy, and radiation for renal cell carcinoma. In addition, she had a decade-long history of fatigue and diffuse pain and numbness, particularly of her lower extremities, treated with non-steroidal anti-inflammatory drugs as needed.

**Figure 1:**
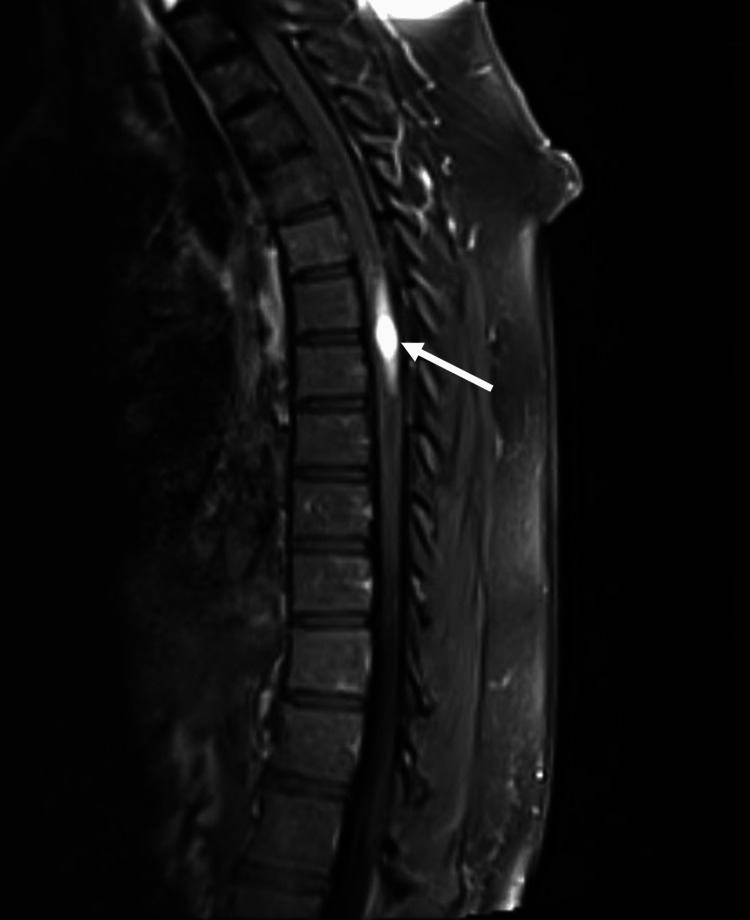
Sagittal MRI T1 sequence with gadolinium contrast demonstrating avidly enhancing intramedullary lesion at T6-7.

**Figure 2:**
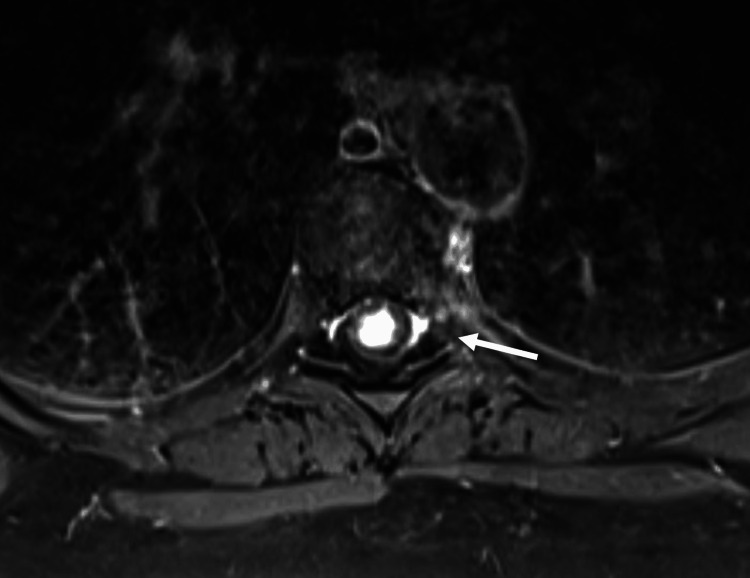
Axial MRI T1 sequence with gadolinium contrast demonstrating the same T6-7 lesion.

**Figure 3:**
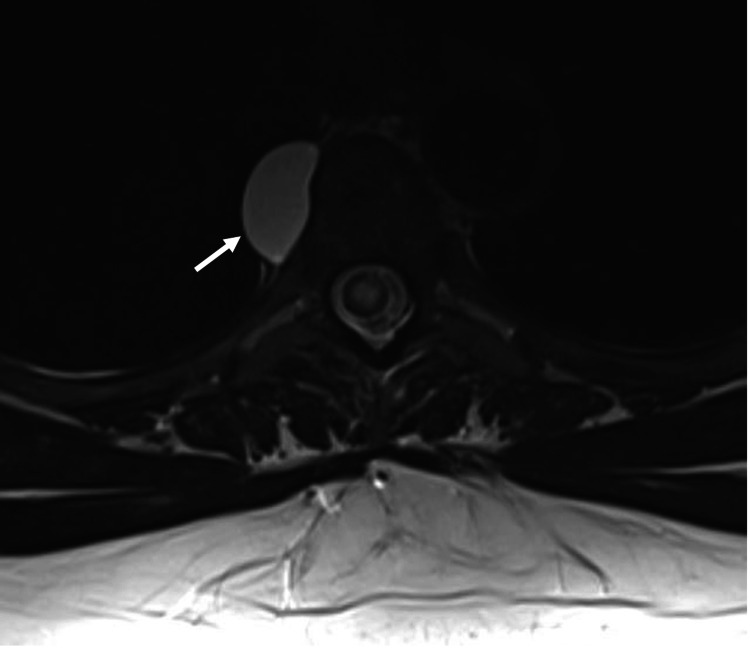
Axial MRI T2 sequence demonstrating the right-sided T5 paraspinal cystic lesion.

Symptomatology and concern for possible cancer recurrence prompted imaging. MRI demonstrated a syrinx spanning the spinal cord to the conus medullaris and a contrast-enhancing, expansile intramedullary thoracic lesion at the level of T6-T7 with a non-enhancing, cystic right paraspinal lesion at the level of T5 (Figures 1-3).

Given its intramedullary location, the T6-T7 lesion was suspicious for primary cord tumors such as astrocytoma and ependymoma, which together comprise the majority of spinal cord tumors. The paraspinal T5 lesion was suspected to be a benign neuro-enteric or duplication cyst, and thus surgical intervention was not indicated. Computed tomography (CT) of the thorax was notable for multiple pulmonary nodules and soft tissue nodularity anterior to the hepatic flexure suspicious for scarring. Given the patient’s history of a kidney neoplasm, a metastatic work-up was completed, and a mesenteric soft tissue nodule biopsy was taken through the abdominal wall. Pathology was consistent with endometriosis, and the patient was instructed to follow up with the gynecology outpatient department.

Five days after presentation, the patient elected to proceed with surgical biopsy of the intramedullary lesion with possible resection. The patient underwent a bilateral, T6-8 total laminectomy with facet preservation to prevent instability and intraoperative neuromonitoring, excisional biopsy, and gross total resection of the intramedullary mass. The lesion was grayish-blue in color, avascular, firm, minimally adherent, and appeared to be encapsulated or pseudoencapsulated. The frozen section of the intramedullary mass indicated presence of tumor cells, favorable for ependymoma. Initial pathology, however, was notable for lymphohistiocytic infiltrate with coagulative necrosis and rare multinucleated giant cells. Stains for spirochete, bartonella, acid-fast bacteria, and fungal elements were negative. The infiltrate was positive for CD3, CD20, PAX5, CD4, CD8, CD68, and CD45, and negative for keratin, GFAP, CD15, and CD30. The mass was negative for carcinoma and ependymoma. Due to the patient’s history of renal cell carcinoma, a keratin stain to rule out metastasis was ordered, and was negative. Immediate postoperative MRI indicated gross total resection of the lesion and significant improvement in thoracic spinal cord edema. In the immediate postoperative period, the patient was unable to ambulate but had improved BLE weakness (strength 5/5 in BLE except 4+/5 left hip flexion, or L HF, and knee extension). Sensation to touch was symmetric and improving in BLE without a clear sensory level, and BLE reflexes were 3+. BLE physiologic clonus was also noted. The patient was discharged with in-home care and a referral to hematology-oncology and neuro-oncology for follow-up.

**Figure 4:**
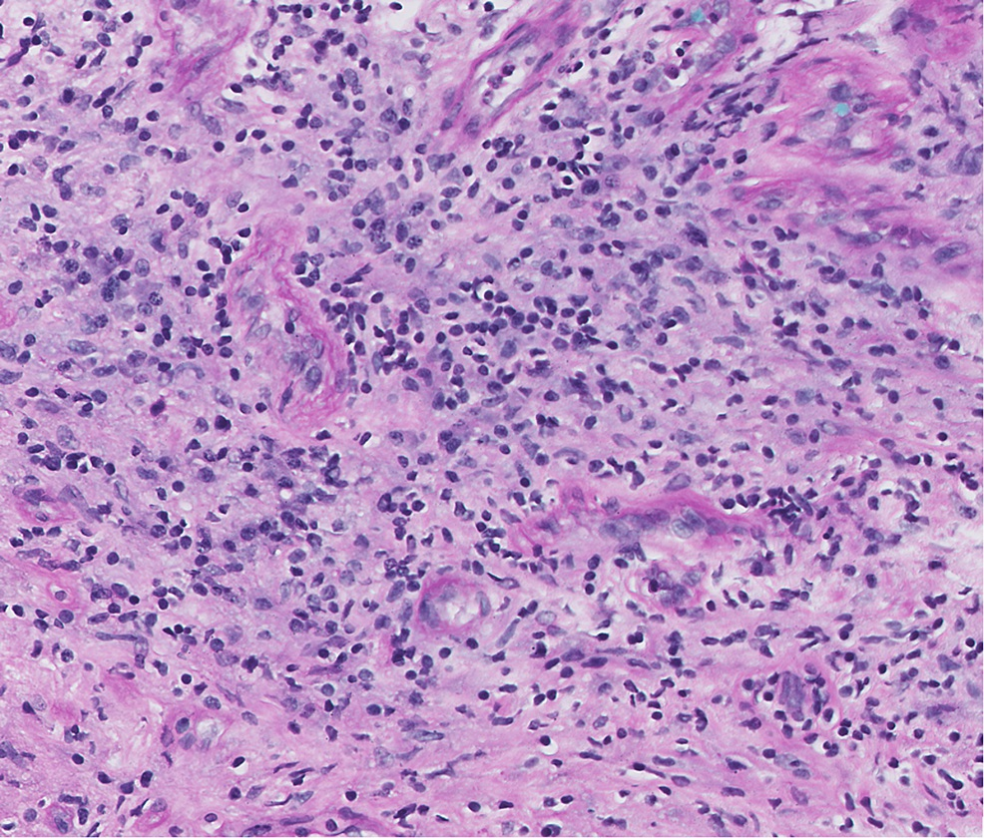
Spine sarcoidosis specimen The permanent H&E-stained section showed a granulation reaction predominated by CD3-positive lymphocytes and macrophages.

A month later, the patient was seen in our neurosurgery clinic for follow-up. She had continued improvement in her LLE weakness (still 5/5 except for 4+/5 strength in left hip flexion and knee extension) and impaired gait, balance, and coordination. Her symptoms of urinary retention, paresthesia, and numbness were resolved. Her one-month postoperative MRI revealed an unchanged T5 cystic lesion, continued resolution of cord edema, and absence of the intramedullary lesion. Final pathology supported a diagnosis of sarcoidosis with a granulation reaction, CD3-positive lymphocytes, and macrophages identified (Figure 4).

**Figure 5:**
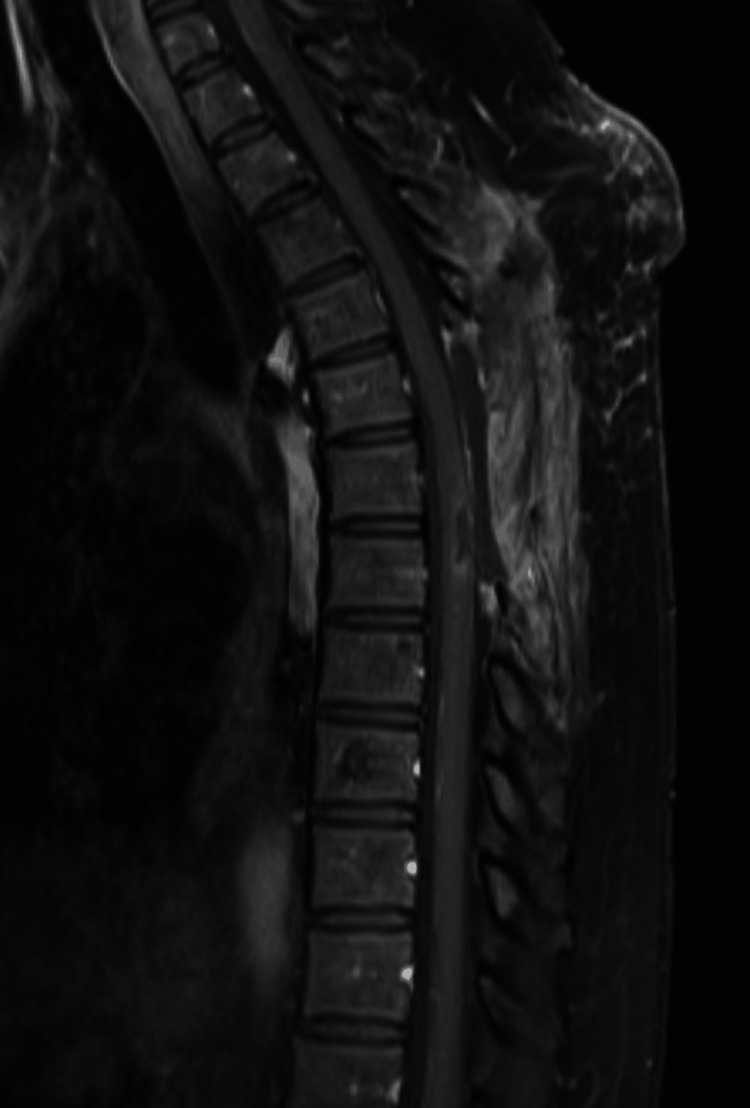
Three-month postoperative sagittal MRI negative for residual enhancement.

A three-month postoperative MRI scan was negative for residual enhancement (Figure 5).

The patient continues to report intermittent pain at the surgical site and is followed up by her primary care consultant for symptom management and monitoring for recurrence.

## Discussion

Literature review

**Figure 6:**
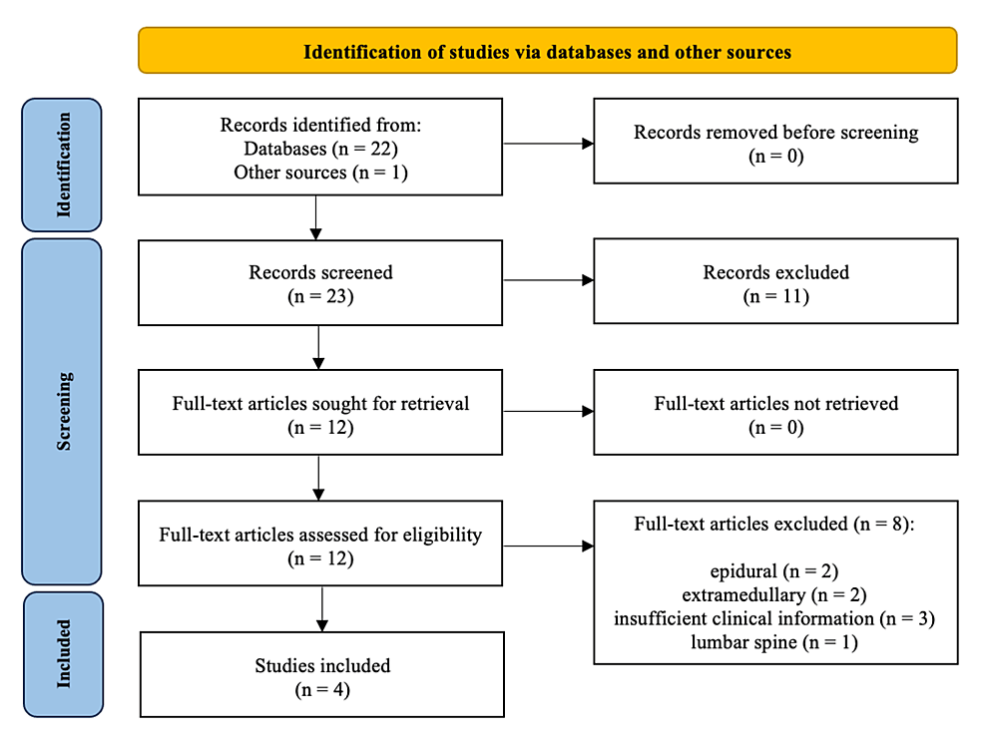
PRISMA figure detailing the intramedullary thoracic spinal sarcoidosis literature review search process. PRISMA, Preferred Reporting Items for Systematic Reviews and Meta-Analyses

A systematic literature review was completed following the Preferred Reporting Items for Systematic Reviews and Meta-Analyses (PRISMA) guidelines [12]. Electronic databases PubMed, Scopus, Web of Science, Ovid Medline, and Cochrane Database of Systemic Reviews were searched for relevant articles using the keywords “sarcoidosis,” “spine,” “thoracic,” “thoracic spine,” and “intramedullary.” A preliminary screening of articles identified via database searches based on title and abstract was completed (see the PRISMA flow diagram, Figure 6).

The reference lists of candidate articles were also assessed for inclusion, in addition to an independent Google search to identify other suitable articles for inclusion. Articles not written in English were reviewed if an official translation was available. Articles were included if they described case reports or case series of adult patients with intramedullary sarcoidosis of the thoracic spine. Articles including only pediatric patients, systematic reviews, and extramedullary spinal sarcoidosis or spinal sarcoidosis not localized to the thoracic spine were excluded. The abstracts of the identified publications were screened by one author (CS) with any conflicts subsequently discussed and resolved by consensus of two authors (CS and JS). The selected publications underwent full-text screening by the same author (CS) based on inclusion and exclusion criteria. Conflicts were discussed and resolved by consensus of the same two authors (CS and JS). Studies that were selected after full-text review for inclusion and exclusion criteria underwent data extraction and analysis by one author (CS) with a review of final data and analysis from the remaining authors. The following clinical data were extracted from the articles and summarized with descriptive statistics as indicated (mean and range, standard deviation, frequencies, and percentages): age, sex, presenting symptoms, radiographic/anatomic location of spinal sarcoidosis, imaging characteristics, pathology findings, steroid therapy, symptoms after treatment, and length of follow-up. 

Initial search criteria identified 23 published studies, with 12 articles undergoing a full-text review (Figure 6). Of these, eight articles were excluded due to insufficient clinical information or incorrect lesion type or location (extramedullary, epidural, and lumbar cord). A total of four studies were included in the final report, each featuring a single patient (n=4) with intradural, intramedullary thoracic spinal sarcoidosis [13-16]​​​​​.

Clinical Presentation

The mean patient age was 53.8 years ± 11.9 (SD) months (range 40-66 years), and there was only one female patient (25%) (Table 1). All patients were symptomatic at presentation (Table 2). Numbness and weakness were the most common symptoms (75%), followed by paresthesia, urinary retention, and urinary incontinence in two patients each (50%). One patient had a prior history of intrathoracic sarcoidosis treated with steroids [13]. Another patient had no signs of sarcoidosis outside of the spine [16]. In the remaining two patients, one had enlarged submandibular lymph nodes negative on biopsy four years prior, and the other had bilateral hilar enlargement at presentation [14,15].

**Table 1:** Published cases of intramedullary sarcoidosis of the thoracic spine ACE, angiotensin converting enzyme; GD, gadolinium; NR, not reported; UTI, urinary tract infection.

Author, year	Age/sex	Spinal level	Symptoms at presentation	MRI findings			Serum ACE	Pathology findings	Outcome at the time of follow-up	Follow-up (months)
				T1	T2	Gd				
Beros et al., 2008 [15]	40/M	T9-12	Impaired ambulation, loss of temperature sensation, loss of touch sensation, paresthesia, urinary incontinence	Thickened cord	Hyperintense, cord edema	Enhancement	Elevated	Noncaseating granulomas with central epitheliod cells, few Langhans cells, and peripheral lymphocytes	Rapid clinical improvement	2.5
Caneparo et al., 2007 [14]	61/M	T5-T6	Numbness, urinary incontinence	NR	Hyperintense	Enhancement	780 U/l	NR	Unchanged sensory symptoms	12
Duhon et al., 2012 [16]	48/M	T10-T11	Back pain, Lhermitte pain, numbness, paresthesia, weakness	NR	Hyperintense	Enhancement	2 U/l	Perivascular noncaseating granulomas with lymphocytic infiltration	Full strength, mild numbness	18
Wang, 1999 [13]	66/F	T10-T11	Numbness, urinary incontinence, urinary retention, weakness	Normal	Normal	Enhancement	22 IU/l	Noncaseating granulomatous inflammation with perivascular lymphocytic infiltration	Mortality (UTI, sepsis)	1

**Table 2:** Summary of associated symptoms in patients with intramedullary sarcoidosis of the thoracic spine (n=4).

Symptom	n (%)
Numbness	3 (75)
Weakness	3 (75)
Paresthesia	2 (50)
Urinary incontinence	2 (50)
Urinary retention	2 (50)
Back pain	1 (25)
Impaired ambulation	1 (25)
Lhermitte pain	1 (25)
Loss of temperature sensation	1 (25)
Loss of touch sensation	1 (25)

Clinical Work-Up

All patients had MRIs with a T2-weighted sequence. Hyperintensity was noted in all but one case (Table 1). Gadolinium contrast was administered in all cases, with lesion enhancement in each case (100%). All patients had lesions confined to the thoracic spinal cord: T5-T6 in one patient, T10-T11 in two patients, and T9-12 in the final patient. In clinical work-up, all but one patient had cerebrospinal fluid (CSF) analysis. All but one patient had surgical biopsy of the intramedullary thoracic spine lesion [14]. The final patient refused biopsy and had a diagnosis of spinal sarcoidosis based on Zajicek criteria [17]. Granulomas were identified on pathology in each case, all described as noncaseating. Langhans giant cells were only identified in one case, whereas lymphocyte infiltration was described in two cases. Postoperative MRI imaging and findings were available in all cases except the case with patient mortality. Two cases described improvement of systemic sarcoidosis, including regression of enlarged lymph nodes and reduction of bilateral hilar enlargement. Reduction of spinal cord edema and contrast enhancement was identified on postoperative MRI in all three cases with detailed postoperative MRI findings.

Treatment and Follow-Up

Two patients had thoracic laminectomies with surgical biopsy followed by steroid therapy [15,16]. The third patient had a surgical biopsy and steroid therapy [13]. The remaining patient only had steroid therapy [14]. Methylprednisone or prednisone was used in all patients. One patient was initially treated with dexamethasone before progressing to methylprednisone and prednisone treatment [13]. Azathioprine was added to one patient’s treatment regimen following pathology diagnosis with surgical biopsy. However, due to side effects, the patient discontinued the immunosuppressant against medical advice [16]. All patients had a definitive length of follow-up, with death reported in one. The mean follow-up was 10.8 ± 7.8 (SD) months (range 2.5-18 months). The mortality occurred within one month due to sepsis from a urinary tract infection [13]. Significant clinical improvement following steroid therapy was seen in two patients, whereas a third patient had largely unchanged neurological symptoms after eight weeks of steroid therapy.

Discussion

The great mimicker, sarcoidosis is a systemic disease that can affect any organ in the body, though no specific etiology is known [18]. Sarcoidosis of the CNS is rare, with an incidence of up to 10%, but spinal sarcoidosis specifically is rarer [4,9,19]. Within the spine, the cervical region is most commonly affected, followed by the thoracic region, as seen in the present case, and which is the focus of this systematic review [5,20-23]. The available literature on spinal sarcoidosis is limited; thus, the reason for a greater incidence in the cervical spine is unknown. The initial presentation of spinal sarcoidosis can include any symptom referable to the spinal cord: pain, myelopathy, weakness, sensory discrepancy, and bowel, bladder, or sexual dysfunction [23]. In the case presented here, urinary retention and numbness were the initial symptoms, followed by weakness and saddle anesthesia after a week. In the systematic review, weakness and numbness were the most common symptoms, identified in three patients each, as well as in the case reported here.

While sarcoidosis is rare in the CNS overall, and rarer still in the spine specifically, it often occurs concurrently in several organ systems, though typically not identified until a diagnosis of sarcoidosis is made [9,21]. Bilateral hilar lymphadenopathy or other lung involvement is found in 90% of sarcoidosis patients, but was identified in only two thoracic spine patients in this review, and was not present in the case presented here [3]. Isolated spinal cord involvement, as seen in the present case and identified in one case in this systematic review, is found in only 16% of spinal sarcoidosis patients [21].

Spinal sarcoidosis can mimic cervical spondylosis, tuberculosis, neuromyelitis optica, demyelinating disorders, and neoplasms [23]. Definitive diagnosis occurs only on pathology; however, several other diseases can present with noncaseating granulomas. Thus, even definitive diagnosis can be a matter of exclusion. The criteria for diagnosing neurosarcoidosis, as proposed by Zajicek et al. in 1999, stratify the diagnostic likelihood into three categories: definite, probable, and possible. Positive nervous system histology is the main differentiating factor between a “definite” and “probable” diagnosis, while “possible” is in patients with absence of histology, laboratory, or MRI evidence but clinical presentation still suggestive of neurosarcoidosis in the absence of alternative diagnoses [17]. Our patient had positive nervous system histology, as did the three cases identified in the literature review that reported pathology. The fourth review patient was diagnosed without biopsy based on Zajicek criteria.

MRI, as seen in the case presented here and all the systematic review cases, is the choice radiographic study in the diagnosis of spinal sarcoidosis. Leptomeningeal enhancement is the most common imaging finding reported in 40% to 67% of neurosarcoidosis patients and identified in the case presented here [24,25]. Cord atrophy, intraparenchymal enhancement, and cord edema can also be seen on MRI [11]. However, a lack of association between MRI findings and concordant clinical progression hinders its utility in diagnosis and treatment response monitoring [24,26-28]. Thus, in cases of isolated thoracic spinal sarcoidosis, as seen in our patient, pathology via surgical biopsy and radiographic findings support ultimate diagnosis. The inability to rely on systemic sarcoidosis findings and lack of biopsy sites outside the spine render definitive diagnosis particularly difficult. In these cases, surgical biopsy of the thoracic spinal lesion may be warranted to allow for definitive diagnosis and more specific treatment recommendations. However, the risk of surgery should be judged on a case-by-case basis.

Laboratory tests are similarly nonspecific in the diagnosis of spinal sarcoidosis and were not performed in the present case. Serum angiotensin-converting enzyme (ACE) levels were tested in all patients in this literature review. However, comparable to their poor predictive value for systemic sarcoidosis, they are reportedly positive in only 23.5% of patients [17,29]. Lymphocytic pleocytosis and increased protein levels are found in the majority of neurosarcoidosis patients on CSF analysis, though reported in only two studies in this literature review [7,15,16]. ACE concentration in CSF can be used to monitor disease progression and treatment outcomes [7,16]. However, its wide range of sensitivity and lack of specificity question its relevancy as a diagnostic indicator [30]. Still, all but one article in this literature review reported ACE concentration [13].

The treatment of spinal sarcoidosis is derived from the medical therapy used to treat neurosarcoidosis overall. Although only a reported 29% of neurosarcoidosis patients respond to steroid monotherapy, corticosteroids remain the first-line treatment [9,11,21,31]. In spinal sarcoidosis patients with systemic sarcoidosis, steroid monotherapy should be the first-line treatment. In spinal sarcoidosis patients without evidence of systemic sarcoidosis where diagnosis is suspected without a surgical biopsy, a trial of intravenous steroids followed by oral steroids should be considered before surgery, especially if surgery is contraindicated [32]. In cases where the diagnosis of spinal sarcoidosis is not made until after surgical biopsy, as in this case, corticosteroids should be started in the immediate postoperative period. In the systematic literature review, all cases were treated with corticosteroids, with half responding to treatment. Of note, the only case without significant symptom improvement was the case that received only oral prednisone and did not undergo surgery. In the case presented here, the patient was placed on corticosteroids in the immediate preoperative and postoperative periods, with symptom relief. In patients who do not respond to steroid monotherapy, progression to steroid-sparing immunosuppressive drugs, such as methotrexate, cyclophosphamide, azathioprine, hydroxychloroquine, and infliximab, is often considered the next step to avoid prolonged high-dose steroid side effects [10,21,31,33-35]. Only one patient required progression to immunosuppressive drugs but discontinued the drug (azathioprine) against medical advice due to side effects.

Beyond medical therapy, there are few treatment options specific to spinal sarcoidosis. Surgery allows lesion biopsy for diagnosis, but aggressive resection beyond that can lead to disease progression and patient deterioration [36]. All but one case in the literature review had surgery for lesion biopsy. Two had laminectomies with resection in addition to surgical biopsy, and interestingly, also had the greatest symptom improvement. Overall, however, spontaneous symptom remission is not seen in spinal sarcoidosis. Early treatment, especially before the onset of paralysis and other severe neurologic deficits, results in better outcomes and prevention of irreversible gliofibrosis [9,37]. In this systematic literature review of intramedullary thoracic spinal sarcoidosis, two patients had improvement with treatment, one died within a month due to sepsis from a urinary tract infection, and the final patient remained stable. The present patient improved with surgical resection and is on follow-up for disease progression monitoring.

Limitations

The literature on spinal sarcoidosis, specifically isolated to the thoracic spine, is limited, thus hindering the extent to which conclusions can be drawn about the cause of the lower incidence in the thoracic region as compared to the cervical spine, and in general about treatment guidelines for spinal sarcoidosis. The only case identified in our literature review that used solely medical management was also the case with patient mortality. Thus, our ability to draw conclusions on surgical versus medical management in isolated intramedullary thoracic spinal sarcoidosis was limited. Likewise, our patient had acute symptoms necessitating surgical intervention; thus, we were unable to examine the benefit of medical management prior to surgical intervention in our case as well.

## Conclusions

Apart from the case presented here, only one case of isolated intramedullary thoracic spinal sarcoidosis was identified in the literature. The dearth of isolated intramedullary thoracic spinal sarcoidosis cases in the literature suggests a lack of reporting, lack of presenting symptoms, or a true lower incidence in these cases, and emphasizes the need for more research and reporting on isolated intramedullary thoracic spinal sarcoidosis. In our case, acute symptoms due to spinal cord compression warranted surgical intervention. While the only case, of both review and presented, without significant symptom improvement was also the case that did not undergo surgery, more research with larger sample sizes is needed to better understand surgical indications and long-term outcomes following medical versus surgical management. Specifically, greater reporting and long-term follow-up of spinal sarcoidosis cases is necessary to update guidelines on managing spinal sarcoidosis patients.

## References

[1] Iannuzzi MC, Rybicki BA, Teirstein AS (2007). Sarcoidosis. N Engl J Med.

[2] Kumar V, Abbas A, Fausto N, Robbins S, Cotran R, Clarence J (2005). Robbins and Cotran Pathologic Basis of Disease. https://www.ncbi.nlm.nih.gov/nlmcatalog/101214483.

[3] Kumar N, Frohman EM (2004). Spinal neurosarcoidosis mimicking an idiopathic inflammatory demyelinating syndrome. Arch Neurol.

[4] Vinas FC, Rengachary S (2001). Diagnosis and management of neurosarcoidosis. J Clin Neurosci.

[5] Junger SS, Stern BJ, Levine SR, Sipos E, Marti-Masso JF (1993). Intramedullary spinal sarcoidosis: clinical and magnetic resonance imaging characteristics. Neurology.

[6] Pirau L, Lui F (2023). Neurosarcoidosis. StatPearls [Internet].

[7] Bradshaw MJ, Pawate S, Koth LL, Cho TA, Gelfand JM (2021). Neurosarcoidosis: pathophysiology, diagnosis, and treatment. Neurol Neuroimmunol Neuroinflamm.

[8] Hayat GR, Walton TP, Smith KR Jr, Martin DS, Manepalli AN (2001). Solitary intramedullary neurosarcoidosis: role of MRI in early detection. J Neuroimaging.

[9] Bradley DA, Lower EE, Baughman RP (2006). Diagnosis and management of spinal cord sarcoidosis. Sarcoidosis Vasc Diffuse Lung Dis.

[10] Varron L, Broussolle C, Candessanche JP, Marignier R, Rousset H, Ninet J, Sève P (2009). Spinal cord sarcoidosis: report of seven cases. Eur J Neurol.

[11] Terushkin V, Stern BJ, Judson MA, Hagiwara M, Pramanik B, Sanchez M, Prystowsky S (2010). Neurosarcoidosis: presentations and management. Neurologist.

[12] Page MJ, McKenzie JE, Bossuyt PM (2021). The PRISMA 2020 statement: an updated guideline for reporting systematic reviews. BMJ.

[13] Wang PY (1999). Spinal cord sarcoidosis presenting as an intramedullary mass: a case report. Zhonghua Yi Xue Za Zhi (Taipei).

[14] Caneparo D, Lucetti C, Nuti A, Cipriani G, Tessa C, Fazzi P, Bonuccelli U (2007). A case of sarcoidosis presenting as a non-specific intramedullary lesion. Eur J Neurol.

[15] Beros V, Houra K, Rotim K, Kovac D, Cupic H (2008). Thoracic intramedullary sarcoidosis mimicking an intramedullary tumor. Coll Antropol.

[16] Duhon BS, Shah L, Schmidt MH (2012). Isolated intramedullary neurosarcoidosis of the thoracic spine: case report and review of the literature. Eur Spine J.

[17] Zajicek JP, Scolding NJ, Foster O (1999). Central nervous system sarcoidosis—diagnosis and management. QJM.

[18] Shadid S, ter Maaten JC (2002). Sarcoidosis - a great mimicker. J Intern Med.

[19] Bogousslavsky J, Hungerbühler JP, Regli F, Graf HJ (1982). Subacute myelopathy as the presenting manifestation of sarcoidosis. Acta Neurochir (Wien).

[20] Jaster JH, Dohan FC Jr., Bertorini TE (1997). Solitary spinal cord sarcoidosis without other manifestations of systemic sarcoidosis. Clin Imaging.

[21] Saleh S, Saw C, Marzouk K, Sharma O (2006). Sarcoidosis of the spinal cord: literature review and report of eight cases. J Natl Med Assoc.

[22] Hamasaki T, Noda M, Kamei N, Yamamoto S, Ochi M, Yasunaga Y (2003). Intradural extramedullary mass formation in spinal cord sarcoidosis: case report and literature review. Spine (Phila Pa 1976).

[23] Chen HI, Lang SS, Coyne TM, Malhotra NR, Schuster JM (2013). Intramedullary spinal sarcoidosis masquerading as cervical stenosis. World Neurosurg.

[24] Lexa FJ, Grossman RI (1994). MR of sarcoidosis in the head and spine: spectrum of manifestations and radiographic response to steroid therapy. AJNR Am J Neuroradiol.

[25] Lury KM, Smith JK, Matheus MG, Castillo M (2004). Neurosarcoidosis—review of imaging findings. Semin Roentgenol.

[26] Shah R, Roberson GH, Curé JK (2009). Correlation of MR imaging findings and clinical manifestations in neurosarcoidosis. AJNR Am J Neuroradiol.

[27] Ahn SW, Kim KT, Youn YC, Kwon OS, Kim YB (2011). Isolated spinal cord neurosarcoidosis diagnosed by cord biopsy and thalidomide trial. J Korean Med Sci.

[28] Koike H, Misu K, Yasui K, Kameyama T, Ando T, Yanagi T, Sobue G (2000). Differential response to corticosteroid therapy of MRI findings and clinical manifestations in spinal cord sarcoidosis. J Neurol.

[29] Hoitsma E, Faber CG, Drent M, Sharma OP (2004). Neurosarcoidosis: a clinical dilemma. Lancet Neurol.

[30] Ungprasert P, Carmona EM, Crowson CS, Matteson EL (2016). Diagnostic utility of angiotensin-converting enzyme in sarcoidosis: a population-based study. Lung.

[31] Lower EE, Broderick JP, Brott TG, Baughman RP (1997). Diagnosis and management of neurological sarcoidosis. Arch Intern Med.

[32] Bhagavati S, Choi J (2009). Intramedullary cervical spinal cord sarcoidosis. Spinal Cord.

[33] Sharma OP (1998). Effectiveness of chloroquine and hydroxychloroquine in treating selected patients with sarcoidosis with neurological involvement. Arch Neurol.

[34] Pettersen JA, Zochodne DW, Bell RB, Martin L, Hill MD (2002). Refractory neurosarcoidosis responding to infliximab. Neurology.

[35] Sollberger M, Fluri F, Baumann T, Sonnet S, Tamm M, Steck AJ, Brutsche M (2004). Successful treatment of steroid-refractory neurosarcoidosis with infliximab. J Neurol.

[36] Day AL, Sypert GW (1977). Spinal cord sarcoidosis. Ann Neurol.

[37] Yasui K, Ishigaki S, Koike H (2000). Correlation of magnetic resonance imaging findings and histopathology of lesion distribution of spinal cord sarcoidosis at post-mortem. Neuropathol Appl Neurobiol.

